# Sperm transfer through hyper-elongated beetle penises – morphology and theoretical approaches

**DOI:** 10.1038/s41598-019-46211-x

**Published:** 2019-07-15

**Authors:** Yoko Matsumura, Jan Michels, Hamed Rajabi, Tateo Shimozawa, Stanislav N. Gorb

**Affiliations:** 1 Department of Functional Morphology and Biomechanics, Zoological Institute, Kiel University, Am Botanischen Garten 1–9, D-24118 Kiel, Germany; 2 Hokkaido University, Sapporo, 060-0808 Japan

**Keywords:** Sexual selection, Biomechanics

## Abstract

Many insects possess a hyper-elongated intromittent organ with a diameter of only a few micrometers. Using morphological and theoretical approaches, we investigated the biomechanics of sperm transfer through such organs by calculating (1) how far and how fast sperm could fill in the penis by capillary action, (2) how much capillary pressure is generated in the penis, and (3) how much pressure is needed to pump sperm out of the penis. The results enabled us to propose the following hypotheses: (1) penile filling basically occurs by capillary action, and (2) sperm transport to females occurs by contracting the sperm pump muscles or by active propulsion of spermatozoa. Potential experimental approaches to test these hypotheses are discussed.

## Introduction

Transferring fluids through a very slender duct does not seem to be an easy task for small animals. However, extremely elongated structures for fluid transfer are ubiquitous. Examples are elongated mouthparts of fluid-drinking insects, which are used for nectar uptake and blood-sucking. The morphology/behavior^1–5^, electrophysiology^6,7^, and mechanics^8–13^ of such mouthparts have been well investigated. According to the literature, the mutually non-exclusive mechanisms for fluid drinking are (1) active suction, (2) capillary suction, and (3) viscous dipping, the latter enabling animals to suck a relatively viscous fluid by dipping their mouthparts in that fluid (summarized in Kim *et al*.^14^).

Fluid transfer through a slender tube also occurs during copulation in many species of a wide range of taxa with a hyper-elongated intromittent organ^15,16^. In insects, those hyper-elongated intromittent organs are very slender. In the extreme case of the leaf beetle species *Lema coronata*, the intromittent organ reaches a length of approximately 10 mm with a diameter of only 2 µm^17^. Nevertheless, these structures are solely used as sperm transferring organs^18–23^, with one exception of a zorapteran species, whose elongated intromittent organ is plausibly used only for sperm displacement^15^. However, the mechanisms of sperm transfer through such structures remain unexplored.

Considering sperm transfer through a hyper-elongated intromittent organ, when males only push sperm actively towards females during copulation, the pushing force should rise with the aspect ratio (length/diameter ratio) of the intromittent organ. Otherwise, the sperm transfer process could take an extraordinarily long time. However, previous observations showed that the duration of copulation in leaf beetles with and without a hyper-elongated intromittent organ is comparable^17,24–26^.

In this study, we carried out morphological investigations and theoretical calculations that aimed at answering the following questions: (1) how do males fill a slender duct with sperm and (2) how do males push the sperm out of a slender duct? Intromittent organ morphology was investigated, and detailed geometric analyses of the intromittent organ were carried out. Since narrower tubes generate a capillary pressure that generally causes ascent and suspension^27^, the contribution of capillary action to the filling of the intromittent organ with sperm was taken into account. Based on the geometric data, we calculated (i) how far and how fast the sperm can move only via capillary action, (ii) how much pressure is generated by capillary action, and (iii) how much force is needed to push a certain amount of sperm into a female within a certain period of time. In addition, we counted the number of spermatozoa transferred during mating in *Lema coronata*, whose intromittent organ was the smallest among the studied species. Based on our results and literature data on analogous fluid drinking mechanisms of insects, we propose possible sperm transfer mechanisms for the studied species and suggest relevant future experiments.

## Systematics and reproductive morphology of the studied species

**Figure 1:**
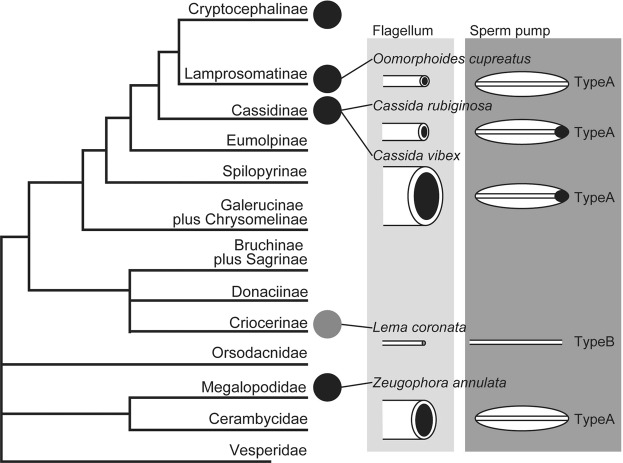
Phylogenetic positions of the studied species and diagrams of the relative flagellum sizes and ejaculatory duct types (type (**A**): with a sperm pump, type (**B**): without a sperm pump). The circles on the cladogram indicate that some species have an extremely elongated intromittent organ, called flagellum. The black color corresponds to A in Fig. 2, and the gray color corresponds to B in Fig. 2. The black ellipses of the sperm pump scheme depict sclerites (see results for further information). The cladogram was modified from Gómez-Zurita *et al*.^65^.

**Figure 2:**
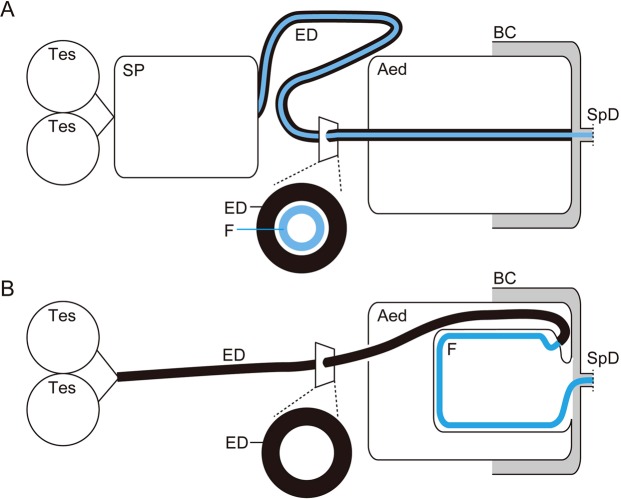
Schematics showing the two different types of the hyper-elongated flagellum found in leaf beetles. (**A**) The ejaculatory duct is elongated, and the hyper-elongated structure, called flagellum, is in the lumen of the duct^25^. (**B**) A specialized pocket for storing the flagellum exists in an aedeagus, and the flagellum is a cuticular prolongation of the end of an ejaculatory duct^35,36^. Abbreviations: Aed, aedeagus; BC, bursa copulatrix; ED, ejaculatory duct; F, flagellum; SP, sperm pump; SpD, spermathecal duct; Tes, testis.

In this study we focused on representative chrysomeloid species. One species of Megalopodidae and four species of Chrysomelidae, all together belonging to four different subfamilies and including two species from one genus, were analyzed (Fig. 1). Four of the studied species (i.e., *Oomorphoides cupreatus*, *Cassida rubiginosa*, *C*. *vibex*, and *Zeugophora annulata*) are fundamentally similar in their reproductive morphology (Figs 1 and 2, type A), which is composed of (1) a long ejaculatory duct^28–30^, (2) an elongated intromittent organ, hereafter called flagellum, which is situated in the lumen of the ejaculatory duct^25^, and (3) a swollen basal part of the ejaculatory duct, which is called either sperm pump^29^ (we adopted this term because of its common use in entomology^31–34^), proximal region^28^, or ejaculatory sac^30^. Another studied species (i.e., *Lema coronata*) has a non-homologous flagellum (Figs 1 and 2, type B), which is a prolongation of the sclerites of the internal sac^35,36^. The swollen part of the ejaculatory duct does not exist there^29,30,37^. In both types, the flagellum is inserted into a female spermathecal duct during copulation to transfer sperm^22,25,38^.

## Results

### Morphology

**Figure 3:**
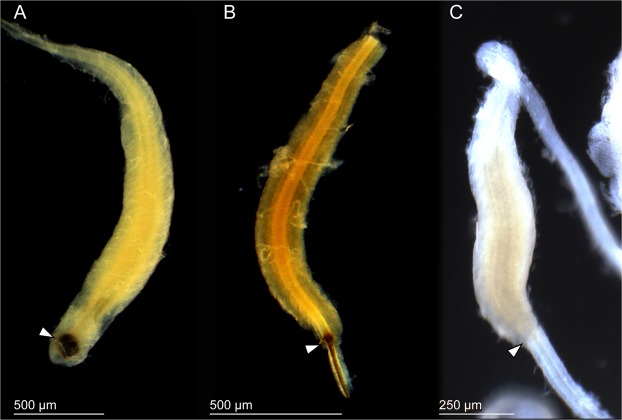
Bright-field micrographs of sperm pumps. (**A**) *Cassida vibex*. (**B**) *Oomorphoides cupreatus*. (**C**) *Zeugophora annulata*. The arrow heads in A and B denote the location of sclerites. The upper side of the sperm pump is the side connected to the testes, and the lower side is connected to the flagellum.

As graphically summarized in Figs 1 and 2, two different types of male reproductive systems were observed in the studied leaf beetles. While the male reproductive system of *Lema coronata* does not feature a sperm pump, those of all other studied species are of type A and include such a sperm pump (Fig. 3), which is a swollen part of the ejaculatory duct and is situated at the basal area of the duct. A sclerotized and strongly melanized sclerite was observed at the proximal part of the sperm pump in *Cassida rubiginosa* and *C*. *vibex*, and a less developed pump was found in *Oomorphoides cupreatus*, but not in *Zeugophora annulata* (Fig. 3, arrow heads).

**Figure 4:**
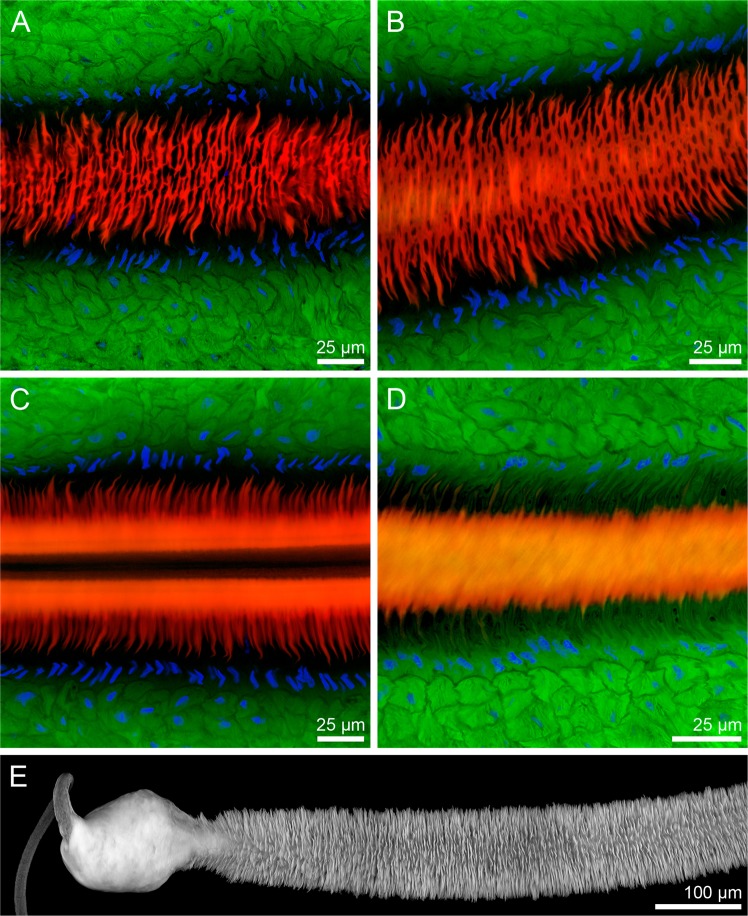
Confocal laser scanning micrographs showing structures of *Cassida rubiginosa* sperm pumps. (**A**–**D**) 0.8-µm-thick optical sections through different horizontal layers of a sperm pump. Red = chitinous structures; green = f-actin cytoskeleton; blue = cell nuclei. (**E**) Maximum intensity projection revealing the chitinous structures (shown in grey) of a section of a macerated sperm pump.

**Figure 5:**
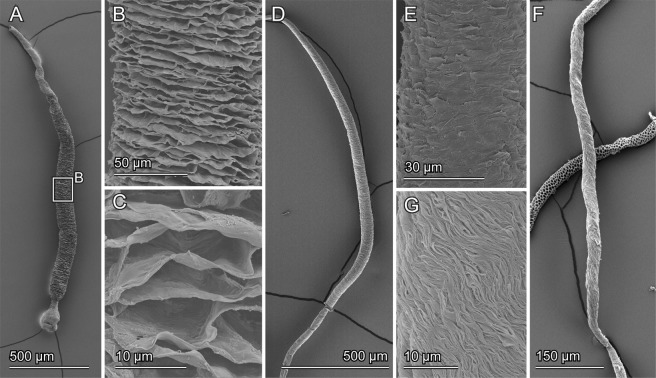
Scanning electron micrographs of sperm pumps, the muscles of which were macerated with potassium hydrate and completely removed. The basal end corresponds to the upper side in all micrographs. (**A**–**C**) *Cassida rubiginosa*. (**D**,**E**) *Oomorphoides cupreatus*. (**F**,**G**) *Zeugophora annulata*. (**A**,**D**,**F**) Whole sperm pumps. (**B**–**E**) Enlarged parts of the middle area of the sperm pumps showing the details of corresponding surfaces.

**Figure 6:**
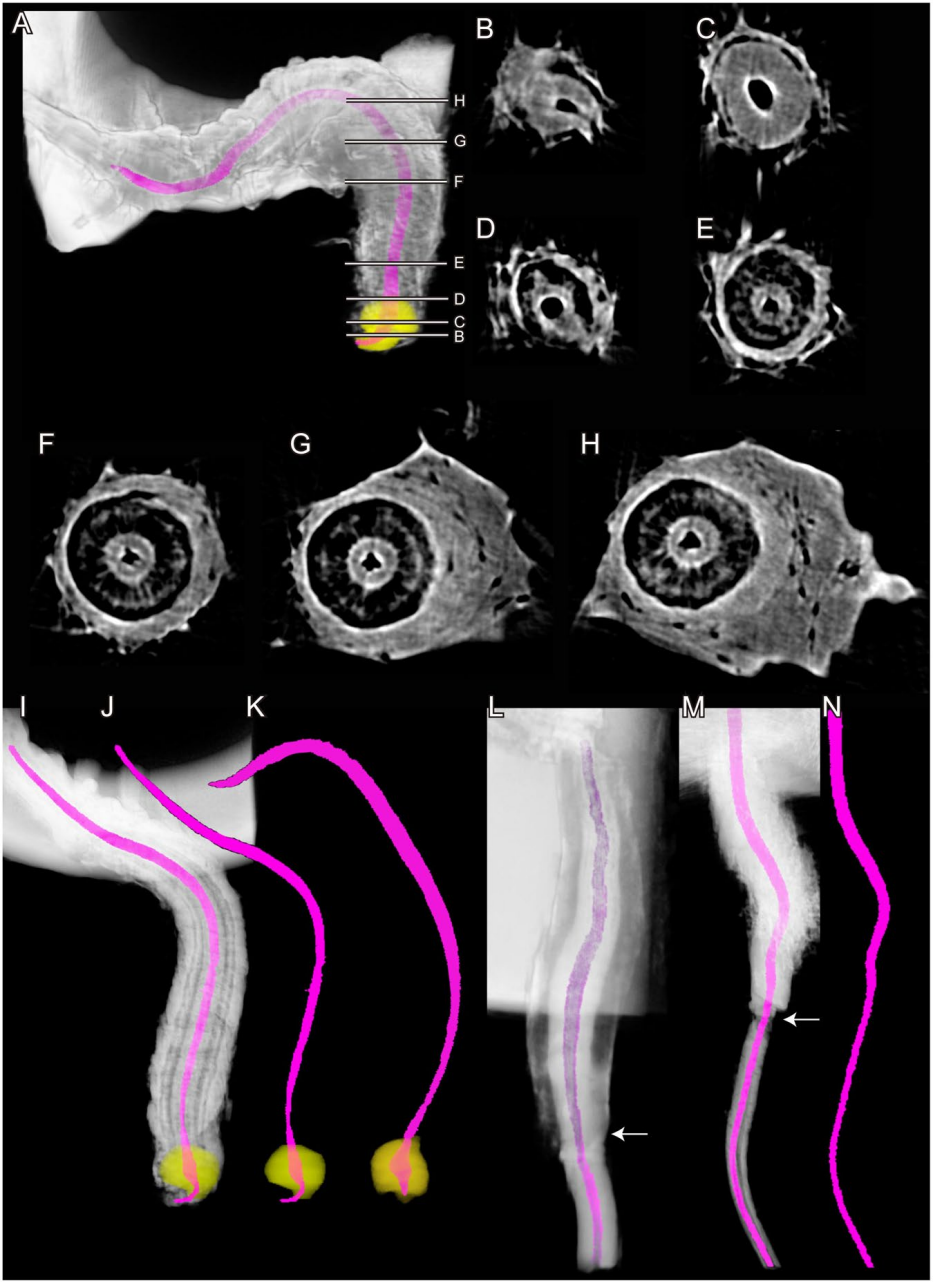
3D reconstructed images of the sperm pump. The lumen of the sperm pump is highlighted in pink, and sclerites in *Cassida* species are colored yellow. (**A**–**J**) *Cassida rubiginosa* (**A**–**H** and **I**,**J** are from two different individuals). (**K**) *Cassida vibex*. (**L**) *Oomorphoides cupreatus*. (**N**,**O**) *Zeugophora annulata*. (**B**–**H**) Cross sections of the sperm pump; the labels of each image correspond to those in A. The arrows in L and M indicate the demarcation of the sperm pump and the rest of the ejaculatory duct.

Using confocal laser scanning microscopy (CLSM), a detailed analysis of the sperm pump of *C*. *rubiginosa* was carried out. We found that well-developed circular muscles (green in Fig. 4) surround the epidermal (whose nuclei are shown in blue in Fig. 4) and cuticular layers (red in Fig. 4). The cuticular part of the sperm pump features a large number of frills in the two *Cassida* species (Figs 4 and 5), while it is more or less smooth in both *O*. *cupreatus* and *Z*. *annulata* (Fig. 5). Within the type A species, differences in the shape of the sperm pump lumen were also found. In *C*. *rubiginosa* and *C*. *vibex*, the lumen size becomes larger at the proximal part of the sperm pump where the sclerite is situated (Fig. 6A–K). In contrast, in *O*. *cupreatus* and *Z*. *annulata*, the lumen size is relatively constant within the sperm pump but slightly tapers toward the flagellum (Fig. 6J–O).

**Figure 7:**
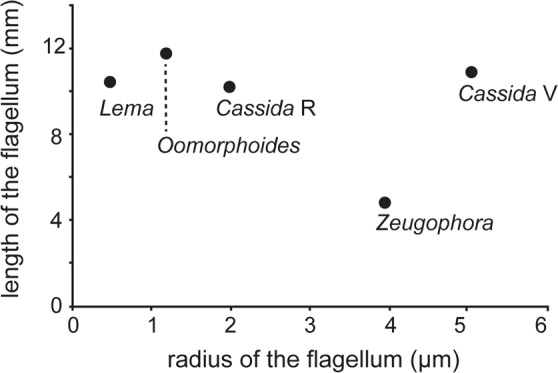
Relationships between the flagellum lengths and the outer radii among the studied species. The generic names on the data points indicate the corresponding studied species. *Cassida* R and *Cassida* V denote *Cassida rubiginosa* and *C*. *vibex*, respectively.

**Figure 8:**
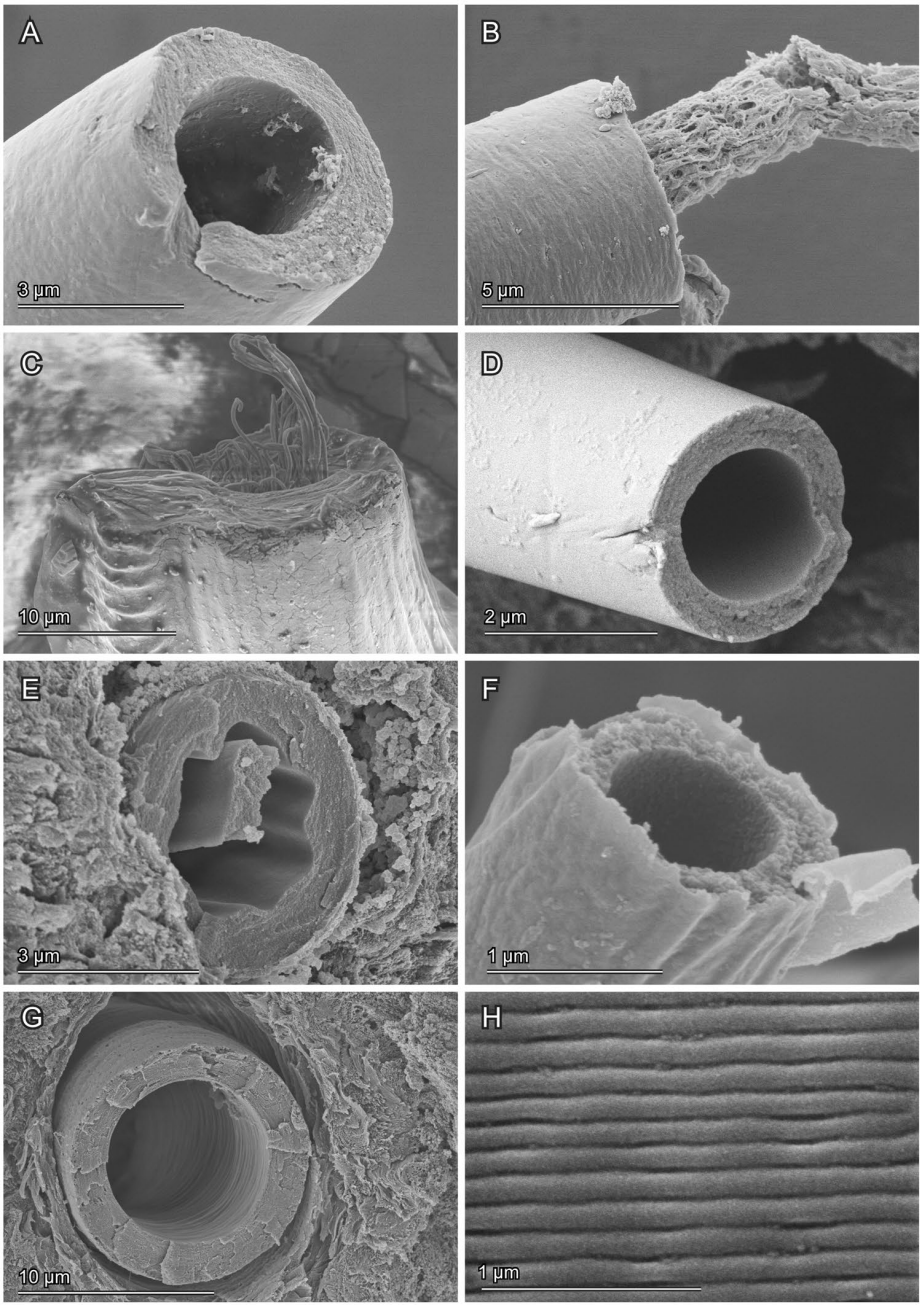
Scanning electron micrographs showing cross sections of flagella from different species. Dried sperm debris protrudes from the flagellum sections in (**B**, **C** and **E**). (**A**,**B**) *Cassida rubiginosa*. (**C**) *Cassida vibex*. (**D**,**E**) *Oomorphoides cupreatus*. (**F**) *Lema coronata*. (**G**,**H**) *Zeugophora annulata*. (**H**) Outer surface of the flagellum.

The flagellum geometry is different among the studied species (Table 1, Suppl. Tables 1–8). In all investigated species, except for *C*. *vibex*, the flagellum has an apex that is narrower than its base. The diameter and the wall thickness of the flagellum are very different among the species despite the comparable flagellum lengths, except for the flagellum of *Z*. *annulata* (Table 1). There is no correlation between the measured flagellum diameter and the measured flagellum length among the studied species (Fig. 7). *L*. *coronata* has the flagellum with the smallest diameter within the studied species. In this species, the inner diameter of the flagellum is less than 1 µm. The largest flagellum diameter was observed in *C*. *vibex*, which has an inner diameter of more than 10 µm. In the studied species, the cross-sectional shape of the flagellum is generally circular (Fig. 8), except for *O*. *cupreatus*, whose flagellum cross section is mainly circular (Fig. 8D) but is sometimes undulated (Fig. 8E). Parallel micro-ridges, arranged perpendicularly to the longitudinal axis, were observed on the inner surface of the flagellum of *Z*. *annulata*. This species also has very regularly arranged ridges oriented along the flagellum axis on the outer surface (Fig. 8H).

**Table 1:** Geometrical measurements of the flagellum in the studied species.

	Body length (mm)	Flagellum length (mm)	Wall thickness (µm)/Outer diameter (µm)				
			Basal	Sub-basal	Middle	Sub-apical	Apical
*Cassida rubiginosa*	ca. 6.5^1,†^	10.2^1^	1.17/6.15 (1)^2^	1.09/6.05 (2)^2^	1.05/6.05 (2)^2^	1.05/5.30 (2)^2^	0.84/4.88 (1)^2^
*Cassida vibex*	5.9^3^	10.9^3^	2.77/13.5 (2)	3.37/16.2 (2)	3.97/18.0 (2)	4.58/18.0 (1)	3.60/18.1 (2)
*Oomorphoides cupreatus*	3.3 (4)	11.7 (1)	1.08/4.98 (2/1)	1.01/5.43 (2)	0.64/3.64 (2)	0.57/3.42 (1/2)	0.72/4.19 (2)
*Lema coronata*	5.0–6.0^4^	10.4^5^	0.80/3.43 (1)	0.30/1.59 (1/2)	0.28/1.55 (2)	—	0.28/1.60 (1/2)
*Zeugophora annulata*	4.3 (3)	4.8 (1)	1.91/12.3 (1)	—	2.07/12.0 (1)	—	1.89/11.0 (1)

Averages are shown, when more than one individual were measured. The numbers in parentheses indicate the numbers of measured individuals. Individual measurements are available in the Supplementary Tables 1–8.

^1^Filippov *et al*.^66^; ^2^Matsumura *et al*.^67^; ^3^Matsumura *et al*.^25^; ^4^Kimoto & Takizawa^68^; ^5^Matsumura & Suzuki^37^; ^†^Originally the value was reported as 10.36 mm. However, we found that the microscope, we used for the measurement in the original paper (Filippov *et al*.^66^) was temporality misarranged, and the measurement based on microscopic images provided 1.6 times bigger values than the real one.

### Theoretical calculations of the sperm transfer

To better understand the sperm transfer mechanism, we performed a theoretical analysis. Due to the lack of data on the physical properties of the sperm and the flagellum cuticle, we first made some assumptions. We used the terms ‘flagellum’ and ‘sperm’ instead of ‘hypothetical flagellum’ and ‘hypothetical sperm’ below, although the properties of glass and water were used for them, respectively. In addition, for simplicity, these calculations were performed by using the radius of the middle region of the flagellum and by assuming a homogenous radius along the flagellum length.

**Figure 9:**
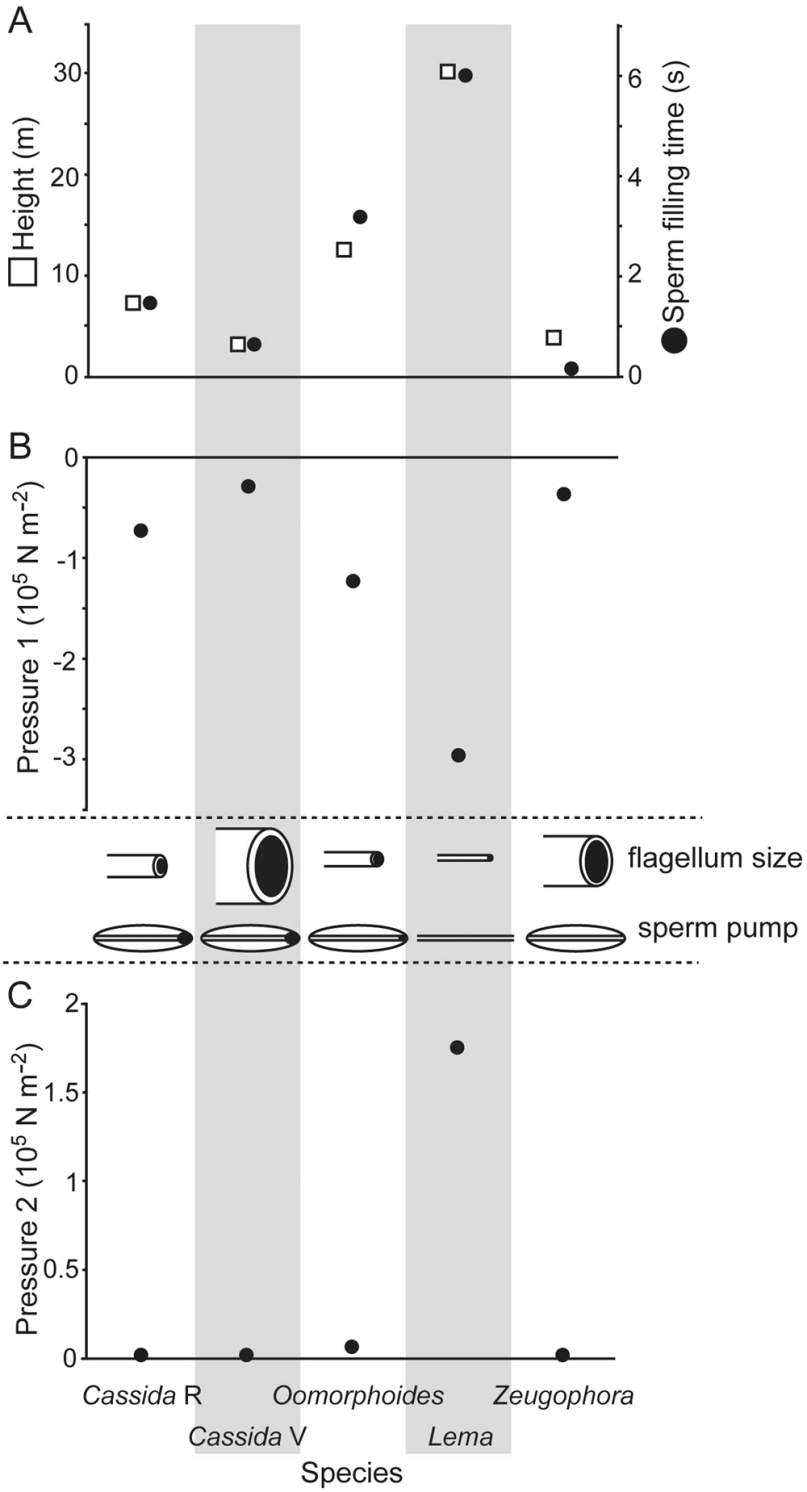
Theoretical calculations of the sperm transfer. These calculations were carried out under the assumptions that (1) the sperm has the same physical properties as those of water at 20 °C and (2) the flagellum is made of perfectly clean glass. The generic names plotted on the x-axis correspond to the species studied, and *Cassida* R and *Cassida* V denote *Cassida rubiginosa* and *C*. *vibex*, respectively. The schemes drawn between the graphs B and C represent the relative sizes of the flagella and the types of the sperm pump of each species. (**A**) Meniscus heights (in meter) and the durations of filling the flagellum with the sperm (in second). (**B**) Magnitude of the negative pressures generated by the capillary effect at the interface of sperm and air. (**C**) Magnitude of the pressures necessary to push the sperm out.

The heights of a semen meniscus in the flagella were estimated using equation (9) (Fig. 9A, Suppl. Table 9). The estimated sperm meniscus heights in the flagellum are all at the meter scale. The lowest height with a value of 2.96 m was estimated for *C*. *vibex*, which shows the largest flagellum diameter (Fig. 9A).

The sperm filling time, which is the time the filling of the flagellum lasts, was calculated using equation (7). This time is in the range of seconds (Fig. 9A, Suppl. Table 9). In the longest case, it takes 6.03 s to fill the flagellum with sperm in *L*. *coronata*, which has the smallest flagellum diameter. In the shortest case, in *Z*. *annulata*, the sperm filling process takes only 0.16 s (Fig. 9A).

The pressure resulting from the capillary action was calculated using equation (1) (Fig. 9B, Suppl. Table 9). The largest negative pressure, 294,383 Nm^−2^, was observed in *L*. *coronata*, which has the smallest flagellum diameter. In other species with larger flagellum diameters, the pressures were much lower ranging between 28,970 and 123,491 Nm^−2^.

In all the calculations mentioned above, the contact angle between the flagella and the sperm was assumed to be 0°. As a comparison, we repeated our calculations with an assumption of a contact angle of 89°, as another extreme situation. These calculations indicate that the height of the semen meniscus in the flagellum is 57.3 times smaller than that calculated before, with *C*. *vibex* featuring the smallest height with 5.17 cm. The sperm filling time is 57.3 times longer than that calculated before, with the longest time of 5.76 min being calculated for *L*. *coronata*. Furthermore, the negative pressures resulting from the capillary action are 57.3 times smaller than those calculated before, with *L*. *coronata* having the largest negative pressure of 5,137.59 Nm^−2^.

To calculate the pressure that is necessary to push the sperm out of the flagellum, the volumetric flow rate of the sperm transferred to females during copulation was calculated based on the number of spermatozoa transferred during a single copulation in *Lema coronata* (2,662 ± 1,245; N = 33). The volume of the transferred sperm was estimated to be 788,207.85 μm^3^ (using equation (8) due to the flagellum geometry). The copulation in this species lasts for 33 min on average^17^, and the volumetric flow rate was calculated to be 3.98 × 10^−16^ m^3^ s^−1^. The pressure required to pump the sperm out of the flagellum was calculated based on this value. The calculated pressures are shown in Fig. 9C (see in detail Suppl. Table 9). In *L*. *coronata*, the calculated pressure is considerably higher than the pressures calculated for the other species (175,741 vs. 17.3–6,122 Nm^−2^).

## Discussion

This study confirms that some chrysomeloid species with a hyper-elongated flagellum have a pump-like structure (sperm pump) featuring stout circular muscles. In addition, there are strong geometric variations in the flagellum among species. Such geometric variations are suggested to alter sperm transfer mechanisms among the studied species as discussed below.

### Flagellum filling

The estimated meniscus height is much larger than the flagellum lengths, and the estimated time needed to fill the flagellum with sperm is on the time scale of seconds. This means that the flagellum lumen can be filled with sperm only by the capillary effect resulting from the flagellum geometry. The flagellum filling process does not necessarily occur immediately before every copulation because the flagellum is a part of the entire reproductive system and is basically always in contact with sperm. However, it is known that non-matured males in *Lema coronata* expose their flagellum before mating^39^ and that males of the lucanid species *Lucanus cervus* emerge with an exposed flagellum^40^. Therefore, the flagellum faces, at least in some cases, the risk of desiccation, and then an automatic and consistent sperm filling is necessary.

Our calculations showed that the capillary action generates a high pressure in the flagellum (e.g., in *L*. *coronata*: −294,383 Nm^−2^ = −0.29 MPa with the assumption of ${\rm{\theta }}=0^\circ$). Insect cuticle, of which the flagellum is composed, has a yield strength ranging from 37–85 MPa^41–43^. This strength is apparently much higher than the highest calculated capillary pressure, which suggests that there is no risk of material failure in the flagellum due to the capillary pressure.

Here, we used some simplified assumptions in our theoretical measurements (e.g., the contact angle ${\rm{\theta }}=0^\circ$ between the sperm and flagellum). In general, sperm contains proteins in addition to spermatozoa (summarized in Werner & Simmons^44^; Orr & Brennan^45^). A protein-rich fluid is demonstrated to feature a contact angle of nearly 0° even on a hydrophobic surface^46^. Therefore, the contact angle of 0° is not a completely unrealistic assumption. Moreover, as a comparison, we recalculated with another extreme assumption of a contact angle of 89°, and the results did not change our discussion points. For a more accurate estimation, the physical properties of the sperm and the inner surface of the flagellum from variable species ideally need to be characterized. In a few recent studies, the viscosity of small volumes of liquids from small insects was successfully measured (for feet secretions^47^ and hemolymph^48^). Although the semen is a mixture of liquids and movable spermatozoa, the methods used in those studies could also be applied to measure the properties of the semen. Another possible experiment corroborating our hypotheses would be to visualize semen movement by capitally action, for example, in glass tubes whose surface wettability is changed by ionization.

### Sperm transport

The second step of the sperm transfer, sperm transport to a female, in the two different reproductive system types A and B (Figs 1 and 2) is accomplished by different mechanisms. Our pressure calculations showed that the pressure needed for transferring the sperm is extremely low in the studied species, except for *L*. *coronata* (17.3–6,122 vs. 175,741 Nm^−2^), compared to previously measured stresses of insect muscles (2–80 × 10^4^ Nm^−2^; Goyens *et al*.^49^ and literature therein). The calculated pressures are in the physiological range that insects can generate with their muscles, and it is reasonable to consider that the sperm pump with its circular muscles present in the studied species, except for *L*. *coronata*, can push the sperm out.

We should take into account that the assumptions used for the calculation of the pressure might have resulted in an underestimation of the calculated values due to the following reasons. First, the sperm transferred to a female does not have to feature the same physical properties as water because sperm usually contains many proteins in addition to spermatozoa^44,45^. This assumption can result in the assignment of a lower dynamic viscosity compared to that of the sperm. Second, we estimated the volume of the transferred sperm based on the data obtained from *L*. *coronata*. The number of spermatozoa transferred in this species (2,662 on average) is extraordinarily smaller than that of other beetles, e.g., 21,610 ± 14,216 in *Chelymorpha alternans* (Chrysomelidae: Cassidinae) (also transferred through a hyper-elongated flagellum)^50^, 56,199 ± 4,462 in *Callosobruchus maculatus* (Chrysomelidae: Bruchiinae)^51^, and 132,000 ± 19,200 in *Tribolium castaneum* (Tenebrionidae) (packed in spermatophores)^52^. Two of our studied species, *Cassida rubiginosa* and *Ca*. *vibex*, which are similar to *Ch*. *alternans*, belong to the Cassidinae and likely transfer more spermatozoa than *L*. *coronata*. However, the larger number of transferred spermatozoa does not mean that those species transfer a larger semen volume since the semen volume was not directly measured in both the current and previous studies. Instead, we estimated the semen volume by assuming that due to the flagellum geometry of *L*. *coronata* the spermatozoa are transferred in a line. However, this might not be the case for other species with larger inner diameters of the flagella.

We found that *Cassida* species possess numerous frills made of cuticular layers under the muscles of the sperm pump and a sclerotized bulb at the proximal end of the sperm pump. These frills are absent in *Oomorphoides cupreatus* and *Zeugophora annulata*. They might provide spatial flexibility of the sperm pump for squeezing out the sperm, which could result in larger pumping pressures. An additional difference in the sperm pump between the *Cassida* species on one side and *O*. *cupreatus* and *Z*. *annulata* on the other side was observed in the size of the lumen in the sperm sclerite. Only in the *Cassida* species is the lumen of the sclerite larger than that of the frilled part, which could result in an alteration of the sperm flow. To test the proposed hypotheses, the sperm pump muscles would have to be experimentally stimulated by electro-physiological techniques. In addition, computer-based fluid dynamics simulations would be helpful.

In contradiction to the mechanisms controlled by the sperm pump, it is more difficult to explain the sperm transport in *L*. *coronata*. Once the sperm fills the flagellum, the capillary action does not work anymore. In this species, the estimated pressure is very high in comparison to the other studied species, and no pump-like structure exists, which means that the sperm is (1) actively sucked in by the female reproductive organs, (2) transferred by active propulsion of the spermatozoa themselves, and/or (3) passively transferred by osmotic pressure differences between the sexes. Those options are not mutually exclusive. However, female active sucking is unlikely because the female reproductive system in *Lema* species does not have specialized modifications that can generate pressure^30,37,53^. The only plausible mechanisms are active spermatozoa propulsion, also known as the spermathecal filling mechanism that occurs after sperm transfer in a rove beetle^54^, and the use of osmotic pressure. It is difficult to estimate how much osmotic pressure contributes to the sperm transport because the concentrations and viscosities of the sperm and the female reproductive secretions are unknown. However, it might not be appropriate to explain the sperm transport of *L*. *coronata* only by active spermatozoa motility. Taking into account the velocity of spermatozoa in the rove beetle (47.5 μms^−1^)^54^, the transfer of 2,662 spermatozoa that are 157 μm (see the method) in length should take 146.6 min. However, the sperm transport, in practice, takes only 33 min on average^17^. Sperm motility patterns differ not only among species but also depending on physiological conditions, i.e., *in vivo* vs. *in vitro*^44^. Therefore, either the spermatozoa of *L*. *coronata* are propelled much faster than those of the rove beetle or the osmotic pressure might affect spermatozoa movement as a tailwind. To test this hypothesis, a visualization of the sperm arrangement in the flagellum and sperm kinematic analyses are needed.

### Comparisons with fluid (nectar) drinking mechanisms

The apparent analogy between nectar drinking of butterflies/bees and the above described sperm transfer in beetles suggests possible mechanical similarities. Although their mouthparts are much more complex than the elongated beetle flagellum and composed of several components^1,3,9^, the uptake of fluid in butterflies is also driven at least partly by a capillary effect^8,9^ that is similar to what we propose here. As summarized by Kim *et al*.^14^, capillary action plays a major role in fluid (nectar) drinking even in birds, and it is presumably one of the ubiquitous fluid transfer mechanisms in animals. In butterflies, even if their mouthparts are manually split into separate elements, which means that the mouthparts no longer have a cylindrical straw-like shape, fluid uptake occurs^10^. This fluid uptake is caused by a wettable surface and structural modifications such as changing inter-projection spaces at the nanometer and micrometer scales^9,10^. The beetle flagellum has a relatively smooth inner surface, implying the possibility that the contact angle between the inner surface of the flagellum lumen and the sperm is less than 89°. This could be tested, for example, by staining the inner surface of the flagellum with Nile Red, as previously applied for testing wettability of the mouthparts^9,10^.

Micro-ridges similar to those found in *Zeugophora annulata* were observed on the mouthparts of honeybee workers^12,13^. Honeybee mouthparts have a hairy glossa, which is usually enclosed by another mouthpart, the galea. The glossa is protruded, dipped in nectar, and withdrawn while drinking^12,13^. During this process, the micro-ridges work as a drag reduction system between the glossa and the galea^12,13^. The micro-ridges in *Z*. *annulata* beetle are smaller than those of honeybees (a few micrometers in honeybees, but less than one micrometer in *Z*. *annulata*). However, since the sperm contains a large number of spermatozoa, the smaller but analogous micro-ridges might reduce the friction between the spermatozoa and the flagellum wall. Future studies on the kinematics of spermatozoa and visualizations of the interface between semen and flagellum walls could test this hypothesis.

In some nectar drinking insects, a sucking pump is present in the head capsule. The periodic movements of this pump cause nectar sucking and transfer of the nectar to the gut^3,5,8,9,55^. In our study, we found the sperm pump, which features stout circular muscles, in four out of the five studied species having flagella with relatively large diameters. Both the sucking pump and the sperm pump work actively, but the first one sucks fluid in while the other one pushes fluid out. The direction of the fluid flow could depend on the direction in which the muscle pumping occurs. To further understand the pumping mechanisms, visualizations of the sperm pump movement during sperm transfer could be implemented by applying phase-contrast x-ray imaging, as has been carried out for the fluid uptake process of butterflies^55^.

This study suggests the importance of the geometrical variation of the apparently simple flagellum, which contributes to the flagellum’s functionality. Our assumption that the dynamic viscosity of sperm is the same among species might conceal the diversity of the sperm transfer mechanisms among the studied species. Future studies should focus on investigating the physical properties of the sperm/flagellum, in particular sperm viscosity, and also on testing the hypotheses introduced here.

## Materials and Methods

### Studied animals

We analyzed the five leaf beetle species *Cassida rubiginosa*, *C*. *vibex*, *Oomorphoides cupreatus*, *Lema coronata*, and *Zeugophora annulata*. Fresh samples were available from the two *Cassida* species. These were dissected in phosphate-buffered solution using an Olympus SZX12 stereo microscope (Olympus Corporation, Tokyo, Japan), and then they were fixed with different fixatives depending on the intended purpose as described below. From the other species, we mainly investigated samples of *O*. *cupreatus* and *L*. *coronata* fixed with a mixture of formaldehyde, acetic acid and ethanol (FAE) and preserved in 70% ethanol and of *Z*. *annulata* preserved in 70% ethanol. When necessary, we took stereo micrographs focused in different layers of the preparations and created micrographs with an extended depth of field by focus stacking using a Leica M205A stereo microscope with a Leica DFC420 camera and the software LAS V3.8 (Leica Microscopy GmbH, Wetzlar, Germany). Based on these micrographs, further measurements were performed using the software Fiji^56^.

### Confocal scanning electron microscopy

Ejaculatory ducts of *C*. *rubiginosa* were dissected, fixed and stained for chitin, nucleic acids and f-actin as described earlier^25,57^. In addition, one ejaculatory duct was transferred to a 10% potassium hydroxide solution for one week to macerate the muscles. Subsequently, it was stained for chitin in the same way as the other samples. All specimens were mounted and visualized using a ZEISS LSM 700 confocal laser scanning microscope system and ZEISS Plan-Apochromat objectives with numerical apertures of 0.8 and 1.3 (Carl Zeiss Microscopy GmbH, Jena, Germany) as previously described^25,57^.

### Scanning electron microscopy

To visualize the exoskeleton of the sperm pump, samples preserved in 70% ethanol or fixed with FAE were soaked in a 10% potassium hydroxide solution to macerate the muscles. The structures were kept in the solution from a couple of hours to days depending on sample conditions. Then, they were gradually dehydrated with an ascending ethanol series and dried using a critical point drier (E3100, Quorum Technologies LTD, Kent, UK). The samples were mounted on carbon sheets, sputter-coated with gold-palladium (10 nm) and visualized with a Hitachi S-4800 scanning electron microscope (Hitachi High-Tech. Corp., Tokyo, Japan) at an accelerating voltage of 3 kV.

We also used scanning electron microscopy (SEM) images to measure the diameter and the wall thickness of the different flagella. For this purpose, the flagella were dissected from beetles preserved in 70% ethanol or fixed with FAE and then dried with the method described above. Subsequently the flagella were cut into either three sections (*Z*. *annulata* and *L*. *coronata*) or five sections (the other species). Each section was cut into pieces and mounted on carbon sheets in a way that the cross sections of the flagella were facing upward. The cross sections were visualized with SEM. Based on the resulting micrographs, the diameters and the wall thicknesses of the flagella were measured with the software Fiji. We analyzed two individuals of each species except for *Z*. *annulata*, of which we used only one individual. All basic statistics were calculated with R^58^.

### Micro-computed tomography

To visualize the shape of the sperm pump lumens, sperm pumps dissected from beetles were dried at the critical point (see above). Then, each sample was fixed on a thin-walled borosilicate glass capillary (120 × 1 mm, Hirschmann-Laborgeräte GmbH & Co. KG, Eberstadt, Germany) with a super glue and analyzed using a SkyScan 1172 high-resolution micro-computed tomography system (RJL Micro & Analytic GmbH, Karlsdorf-Neuthard, Germany) with a current of 250 µA and a voltage of 40 kV. The segmentation and processing of the images were performed with the software Amira 5.4 (Visualization Sciences Group, Mérignac, France).

### Quantifying spermatozoa

For the quantification of the spermatozoa transferred through the elongated flagellum, we had to obtain virgin females and make them copulate under lab conditions. *L*. *coronata* was the only species that met these prerequisites^17^. Therefore, we experimentally dealt only with this species. Beetles were collected in Shiga, Japan, in June 2008 and kept in plastic bags in a conventional incubator with the light conditions 16 L/8 D and at 20 ± 0.5 °C. Eggs were collected regularly, and the hatched larvae were kept until they reached adulthood. After pupation, the animals were separated into plastic boxes with respect to gender and kept together with their food plant *Commelina communis* ad libitum. The animals were checked every day.

Thirty-three pairs of approximately two-week-old virgin males and virgin females were prepared to mate. Immediately after they had finished the first copulation, the males and females were placed and kept in a conventional freezer (−20 °C). Later, the females were defrosted by keeping them at room temperature for ca. 5 min., the spermathecal capsules were dissected, and the spermatozoa were counted using a standard method^59,60^. For this, each spermathecal capsule was placed in a 1.5 ml Eppendorf tube and crushed into small pieces with fine tweezers. Then, 300 µl of distilled water were added, and the sperm were homogenized with a IKA MS3 Basic Vortex Mixer (IKA, Osaka, Japan). Fifteen 1 µl-droplets were placed on glass slides and dehydrated by leaving them at room temperature for half an hour. Then, 80% ethanol was dropped on each trace of diluted sperm. After 2 min, the samples were rinsed with tap water and air-dried at room temperature. The sperm was stained with 4% Giemsa stain solution (Merck KGaA, Darmstadt, Germany) for 15 min., rinsed with tap water and dried at room temperature. The samples were observed using a Zeiss Axiophot bright-field microscope (Carl Zeiss Microscopy GmbH), and the number of the spermatozoa in each spot was counted. Based on the results, we estimated the total number of spermatozoa stored in the spermathecal capsule directly after copulation.

### Theoretical calculations of the sperm transfer

Using a theoretical method, we calculated (1) how far and how fast sperm can fill the flagellum as a result of capillary action, (2) how much pressure to fill the flagellum is generated by capillary action, and (3) how much pressure is necessary to pump sperm out of the hyper-elongated flagellum. Due to the lack of data on the physical properties of the sperm and the flagellum cuticle, the theoretical calculations were performed with some assumptions. We assigned the properties of water and glass to the sperm and flagellum wall, respectively. The properties used for the calculations are listed in Table 2.

**Table 2:** The values used for the theoretical calculations.

Parameters	Values	References
*γ*	72.86 mN m^−1^ (water – air at 20 °C)	(Harkins & Brown^69^; Pallas & Harrison^70^)
*θ*	0 ° (water –perfectly cleaned glass)	(Shaughnessy *et al*.^71^), although a dynamic contact angle between glass and water changes chronologically (Engländer *et al*.^72^), for simplicity we assumed that a contact angle does not change.
*ρ*	998.19 kgm^−3^ (at 20 °C)	(Engineering ToolBox^73^)
μ	1.0005 × 10^−3^ Ns m^−2^ (at 20 °C)	(Engineering ToolBox^74^)

The pressure difference at the interface between the filled and empty parts of a tube, known as capillary (or Laplace) pressure, P_c_, can be calculated using the following equation^61^:

1 $${{\rm{P}}}_{{\rm{c}}}=\frac{2{\rm{\gamma }}\,\cos \,{\rm{\theta }}}{{\rm{r}}}$$

In the above equation, γ is the surface tension of the fluid, r is the inner radius of the tube, and θ is the contact angle between the fluid and the tube wall. Assuming that the tube wall is effectively wetted by the fluid, resulting in a contact angle of θ = 0, equation (1) can be simplified, and the capillary pressure (P_c_) is formulated as:

2 $${{\rm{P}}}_{{\rm{c}}}=\frac{2{\rm{\gamma }}}{{\rm{r}}}$$

According to the Hagen-Poiseuille law, the pressure difference between the two ends of a fluid in a narrow cylindrical tube, r  $\ll$  L, causes the volumetric flow Q, which can be obtained from the equation by Kirby^62^:

3 $${\rm{Q}}=\frac{\pi {r}^{4}{\rm{\Delta }}P}{8\mu L}$$

where L is the length of the fluid column, μ is its dynamic viscosity, and ∆P is the total driving pressure acting on the fluid in the tube. Assuming that the fluid displacement occurs only via capillary action, by substituting P_c_ from equation (2) for Δ*P* in equation (3), we can write:

4 $$Q=\frac{{{\rm{\pi }}r}^{3}{\rm{\gamma }}}{4{\rm{\mu }}L}$$

Now, assuming that in the time dt, the surface of the fluid inside the tube is displaced by dL, we can write

5 $${\rm{Q}}\cdot dt={{\rm{\pi }}r}^{2}\cdot dL$$

Substituting equation (4) into equation (5) and integration give

6 $${\frac{L(t)}{2}}^{2}=\frac{r{\rm{\gamma }}}{4{\rm{\mu }}}t+{\rm{C}}$$

where C is an integration constant. Assuming an initial condition of $L(t)=0$ at $t=0$, we can estimate the time required to fill a tube of length L by capillary action using the following equation:

7 $$t=\frac{2{\rm{\mu }}{L}^{2}}{r{\rm{\gamma }}}$$

The above formula was independently derived by Washburn^63^ under similar assumptions.

To calculate the pressure (∆P) transferring a certain amount of fluid using equation (3), the volumetric flow Q has to be calculated. For this purpose, we used the data from *L*. *coronata*, which has the smallest flagellum diameter, because more data are available for this species than for the other studied species. The volume V of the sperm transferred to a female through a copulation was estimated using the following formula with the assumption that spermatozoa are propelled in a line through the extremely narrow duct, as known from a rove beetle^54^:

8 $${\rm{V}}={\rm{\pi }}{r}_{flagellum}^{2}\,{L}_{sperm}{N}_{sperm}$$

where ${r}_{flagellum}$ is the internal radius of the flagellum, ${L}_{sperm}$ is the length of spermatozoa, and ${N}_{sperm}$ is the number of spermatozoa transferred through a single copulation and counted in the present study. Based on a micrograph showing the spermatozoa of *Zophobas confusa* (Tenebrioninae: Tenebrionini) (Fig. 3E in Dias *et al*.^64^), ${L}_{sperm}$ was determined to be 157 μm (data for *L*. *coronata* were not available).

According to the Jurin’s law^27^, the fluid meniscus height can be calculated by:

9 $${\rm{h}}=\frac{2\gamma \,\cos \,\theta }{\rho gr}$$

where $\rho$ is the density of the fluid and *g* is the gravitational acceleration (9.80665 m s^−2^).

## Supplementary information

Supplementary Tables 1-9

## Data Availability

The data are available as supplementary information.
