# Supplementary material for: Sperm transfer through hyper-elongated beetle penises – morphology and theoretical approaches
Source: Sci Rep. 2019 Jul 15;9:10238. doi: 10.1038/s41598-019-46211-x (PMC6629632; doi:10.1038/s41598-019-46211-x)
Supplement: Supplementary file 1 — Supplementary Tables 1-9 [file 41598_2019_46211_MOESM1_ESM.docx]

Supplementary information

**Sperm transfer through hyper-elongated beetle penises – morphology and theoretical approaches**

Yoko Matsumura^1^, Jan Michels^1^, Hamed Rajabi^1^, Tateo Shimozawa^2^, Stanislav N. Gorb^1^

^1^ Department of Functional Morphology and Biomechanics, Zoological Institute, Kiel University, Am Botanischen Garten 1–9, D-24118 Kiel, Germany, ^2^ Hokkaido University, Sapporo, 060-0808, Japan

Supplementary Tables

Table S1. Measurements of the flagellum diameter of *Cassida vibex*, average ± s.d. Unit: µm. In parentheses, the number of measurements from each region is shown.

| Individual | Basal | Sub-basal | Middle | Sub-apical | Apical |
| --- | --- | --- | --- | --- | --- |
| 1 | 10.83 ± 0.96 (2) | 12.82 ± 0.99 (4) | 17.41 ± 1.42 (7) | 17.13 ± 1.90 (4) | 18.43 ± 0.49 (2) |
| 2 | 16.24 ± 0.93 (4) | 19.52 ± 1.88 (4) | 18.48 ± 0.98 (3) | 18.05 ± 1.66 (6) | 17.74 (1) |

Table S2. Measurements of the flagellum wall thickness of *Cassida vibex*, average ± s.d. Unit: µm. In parentheses, the number of measurements from each region is shown.

| Individual | Basal | Sub-basal | Middle | Sub-apical | Apical |
| --- | --- | --- | --- | --- | --- |
| 1 | 2.14 ± 0.47 (4) | 2.62 ± 0.38 (8) | 3.84 ± 0.50 (11) | 4.13 ± 0.60 (11) | 4.03 ± 0.90 (6) |
| 2 | 3.40 ± 0.57 (9) | 4.11 ± 0.42 (7) | 4.09 ± 1.00 (9) | 5.02 ± 0.96 (10) | 3.17 ± 1.37 (2) |

Table S3. Measurements of the flagellum diameter of *Oomorphoides cupreatus*, average ± s.d. Unit: µm. NA: data are not available. In parentheses, the number of measurements from each region is shown.

| Individual | Basal | Sub-basal | Middle | Sub-apical | Apical |
| --- | --- | --- | --- | --- | --- |
| 1 | NA | 5.29 ± 0.30 (2) | 3.98 ± 0.48 (6) | 3.24 ± 0.22 (2) | 3.41 ± 0.59 (3) |
| 2 | 4.98 ± 0.52 (3) | 5.56 ± 0.55 (6) | 3.31 ± 0.30 (4) | 3.60 ± 1.02 (5) | 4.97 (1) |

Table S4. Measurements of the flagellum wall thickness of *Oomorphoides cupreatus*, average ± s.d. Unit: µm. NA: data are not available. In parentheses, the number of measurements from each region is shown.

| Individual | Basal | Sub-basal | Middle | Sub-apical | Apical |
| --- | --- | --- | --- | --- | --- |
| 1 | 1.19 ± 0.02 (2) | 0.96 ± 0.10 (4) | 0.65 ± 0.10 (13) | NA | 0.56 ± 0.07 (6) |
| 2 | 0.97 ± 0.11 (6) | 1.06 ± 0.27 (14) | 0.62 ± 0.15 (7) | 0.57 **±** 0.23 (7) | 0.88 ± 0.03 (2) |

Table S5. Measurements of the flagellum diameter of *Lema coronata*, average ± s.d. Unit: µm. NA: data are not available. In parentheses, the number of measurements from each region is shown.

| Individual | Basal | Sub-basal | Middle | Sub-apical | Apical |
| --- | --- | --- | --- | --- | --- |
| 1 | 3.43 ± 0.28 (2) | 1.53 (1) | 1.52 ± 0.24 (4) | NA | 1.45 ± 0.07 (2) |
| 2 | NA | 1.76 ± 0.07 (4) | 1.58 ± 0.10 (5) | NA | 1.64 (1) |

Table S6. Measurements of the flagellum wall thickness of *Lema coronata*, average ± s.d. Unit: µm. NA: data are not available. In parentheses, the number of measurements from each region is shown.

| Individual | Basal | Sub-basal | Middle | Sub-apical | Apical |
| --- | --- | --- | --- | --- | --- |
| 1 | 0.80 ± 0.18 (2) | NA | 0.29 (1) | NA | 0.28 (1) |
| 2 | NA | 0.30 ± 0.06 (2) | 0.28 ± 0.08 (2) | NA | NA |

Table S7. Measurements of the flagellum diameter of *Zeugophora annulata*, average ± s.d. Unit: µm. NA: data are not available. In parentheses, the number of measurements from each region is shown.

| Individual | Basal | Sub-basal | Middle | Sub-apical | Apical |
| --- | --- | --- | --- | --- | --- |
| 1 | 12.26 ± 0.50 (7) | NA | 12.04 ± 1.62 (3) | NA | 10.99 ± 2.66 (2) |

Table S8. Measurements of the flagellum wall thickness of *Zeugophora annulata*, average ± s.d. Unit: µm. NA: data are not available. In parentheses, the number of measurements from each region is shown.

| Individual | Basal | Sub-basal | Middle | Sub-apical | Apical |
| --- | --- | --- | --- | --- | --- |
| 1 | 1.91 ± 0.18 (14) | NA | 2.07 ± 0.48 (5) | NA | 1.89 ± 0.72 (5) |

Table S9. Theoretical calculations and their corresponding data. The contact angle between the flagella and the sperm was assumed to be 0º.

| Species | Inner radius of the flagellum (µm) | Meniscus height $\mathbf{h}$ (m) | Pressure difference $\boldsymbol{\Delta P}$ (Nm^-2^) resulted from the capillary action | Duration (s) filling the flagellum, not the meniscus height | Pressure difference $\boldsymbol{\Delta P}$ (Nm^-2^), necessary for moving a certain amount of the sperm |
| --- | --- | --- | --- | --- | --- |
| *Cassida rubiginosa* | 1.98 | 7.54 | -73,782.28 | 1.45 | 680.13 |
| *Cassida vibex* | 5.03 | 2.96 | -28,970.18 | 0.65 | 17.27 |
| *Oomorphoides cupreatus* | 1.18 | 12.62 | -123,491.53 | 3.20 | 6,122.35 |
| *Lema coronata* | 0.50 | 30.07 | -294,383.83 | 6.03 | 175,740.98 |
| *Zeugophora annulata* | 3.93 | 3.79 | -37,078.88 | 0.16 | 20.41 |
